# CO_2_ phonon mode renormalization using phonon-assisted energy up-conversion

**DOI:** 10.1038/srep03341

**Published:** 2013-11-26

**Authors:** Nabila Tanjeem, Tadashi Kawazoe, Takashi Yatsui

**Affiliations:** 1 School of Engineering, University of Tokyo, 113-8656 Tokyo, Japan

## Abstract

Molecular dissociation under incident light whose energy is lower than the bond dissociation energy has been achieved through multi step excitation using a coupled state of a photon, electron, and multimode-coherent phonon as known as the dressed photon phonon (DPP). Here, we have investigated the effects of the DPP on CO_2_, a very stable molecule with high absorption and dissociation energies, by introducing ZnO nanorods to generate the DPP. Then, the changes in CO_2_ absorption bands were evaluated using light with a wavelength longer than the absorption wavelength, which confirmed the DPP-assisted energy up-conversion. To evaluate the specific CO_2_ modes related to this process, we measured the CO_2_ vibration-rotation spectra in the near-infrared region. Detailed analysis of the 3*ν*_3_ vibrational band when a DPP source is present showed that DPP causes a significant increase in the intensity of certain absorption bands, especially those that require higher energies to activate.

Carbon dioxide, one of the major greenhouse gases, has been widely studied as an important molecule in Earth's atmosphere and interstellar media. With a view to mitigating the effects of global warming, different approaches to the photoreduction of CO_2_ have been investigated. The bond dissociation energy *E*_diss_ of one CO_2_ molecule has been reported to be more than 7 eV, equivalent to the energy of incident light wavelength of 174 nm (*λ*_diss_). Such a high energy requirement has inspired scientists to investigate photocatalytic reduction of CO_2_ in dissolved liquids. Photocatalytic conversion of CO_2_ to hydrocarbon fuels has been experimented widely and implemented using wide band gap oxides, such as TiO_2_^1^ and ZnO^2^. However, the high energy requirements of this process, its multielectron transfer, and the complex design of the catalysts have limited the efficiency of the photo-electrocatalytic approach. Dissociation of CO_2_ molecules using radio-frequency discharge with maximum energy efficiency of 3% has also been introduced^3^. There have been many reports on the dissociation dynamics of CO_2_ in the gas phase, most of them focusing on two main dissociation paths: a dissociation process CO_2_ + hν → CO(^1^Σ_g_^+^) + O(^1^D) with incident light of wavelength 157 nm (red curve in Fig. 4(a)) and a predissociation process CO_2_ + hν → CO(^1^Σ_g_^+^) + O(^3^P) with deep UV light (174–195 nm, blue curve in Fig. 4(a))^4^. Photodissociation of CO_2_ gas molecules following these two paths with incident light with wavelengths longer than *λ*_diss_ could enable photoreduction of CO_2_ using the solar spectrum from the UV to visible light region, although no report on this process has been published yet. In this research, we performed phonon-assisted energy up-conversion to dissociate CO_2_ molecules by introducing ZnO nanorods. This process has been implemented previously for dissociating gas phase diethylzinc molecules using an optical near field generated around the nanostructure to stimulate multistep excitation via molecular vibrational modes under incident light with an energy less than the bond dissociation energy^5^.

The physics of these nanoscale optical effects has been developed under the assumption of a conventional multipolar quantum electrodynamic Hamiltonian in a Coulomb gauge and of single-particle states in a finite nanosystem^6^. In such a system, fluctuations in the electromagnetic field (e.g., zero-point fluctuations of the vacuum) cause nanomaterials to emit or absorb virtual photons, i.e., the optical near-fields continuously present around illuminated materials. These so-called virtual absorption and emission processes violate the energy conservation law but are consistent with the Heisenberg uncertainty principle, and to take these processes into account, nanomaterial can be considered to be covered with a cloud of virtual photons. Within this framework, a virtual photon can be described as a coupled state of the electron and a real photon (i.e., a free photon). The quasiparticle representing this coupled state has been called a dressed photon (DP), which has a greater amount of energy than a free photon because of energy contributed by the coupled electron. To take advantage of the nanoscale optics, a thorough understanding of the nanoscale material is required. Such nanoscale materials are composed of a crystal lattice, and after a DP is generated at the surface of an illuminated nanoscale particle, its energy can be exchanged with this crystal lattice. Through this exchange, vibrational modes can be coherently excited in the crystal lattice, creating multiple modes of coherent phonon states^7^. Consequently, the DP and a coherent phonon form a coupled state. This state (the dressed photon and phonon, DPP) constitutes a quasi-particle that is generated only when the particle size is sufficiently small that the crystal lattice vibration is excited coherently. In contrast, vibrational modes cannot be excited coherently in bulk materials, and energy is instead dissipated as heat throughout the material, which is heated as a result. Therefore, the DPP has a higher energy than the DP or the incident free photon. Numerous experiments have been reported for which the results were explained by DPP theory, including experiments on photochemical vapour deposition^5^, photolithography^8^, visible-light water splitting^9^, photovoltaic devices^10^, energy up-conversion devices^11^, and Si lasers^12^. In addition, since the DPP is excited only in nanoscale structures, it can be utilized to realize selective etching of nanoscale bumps to achieve atomically flattened substrates^13^. This DPP energy, when absorbed by a molecule adjacent to the nanostructure, allows the molecule to absorb multimode coherent phonons to become excited to a vibrational mode of higher energy. This intermediate excitation of molecules is the key to energy up conversion processes, allowing dissociation with less energy.

Here, we studied the effect of the DPP on CO_2_ molecules. To induce the DPP, we fabricated ZnO nanorod structures with average diameters of 30–50 nm. As the light source for photo-activation of the CO_2_ molecules, we used a YAG laser of λ = 213 nm, which is longer than the maximum absorption wavelength of CO_2_ λ_abs_ = 200 nm. To observe the change in concentration of CO_2_ gas as well as the vibrational excitation of the CO_2_ molecules, we performed infrared spectroscopy. In particular, we measured the absorption spectrum of CO_2_ in the near-infrared region (1430–1452 nm) while irradiating a substrate consisting of ZnO nanorods inside a chamber filled with CO_2_ gas. The infrared absorption in this region mainly results from the asymmetric stretching vibration of CO_2_ molecules and consists of two different transition bands. We analysed the changes in the vibration-rotation spectra due to UV irradiation and the excitation of DPPs. We confirmed that energy was transferred between the two bands (the traditional ‘cold' band and accompanying ‘hot' band) at room temperature under UV irradiation, and this was proved to be a reversible process, as the molecules returned to their initial state after a certain time. However, because of the DPP excitation, significant and irreversible energy transfer occurs, causing the absorption at higher energy ‘hot' bands to increase. We explained this result using the fact that DPP excitation allows molecules to absorb multimode coherent phonons, resulting an average population increase in the high energy bands, in other word, phonon mode renormalization. The result also supports the assumption that DPP excitation permits CO_2_ molecules to absorb light with wavelengths longer than the absorption wavelength (λ = 213 > λ_abs_ = 200 nm).

## Results

### Measurement of CO_2_ absorption spectrum

**Figure 1:**
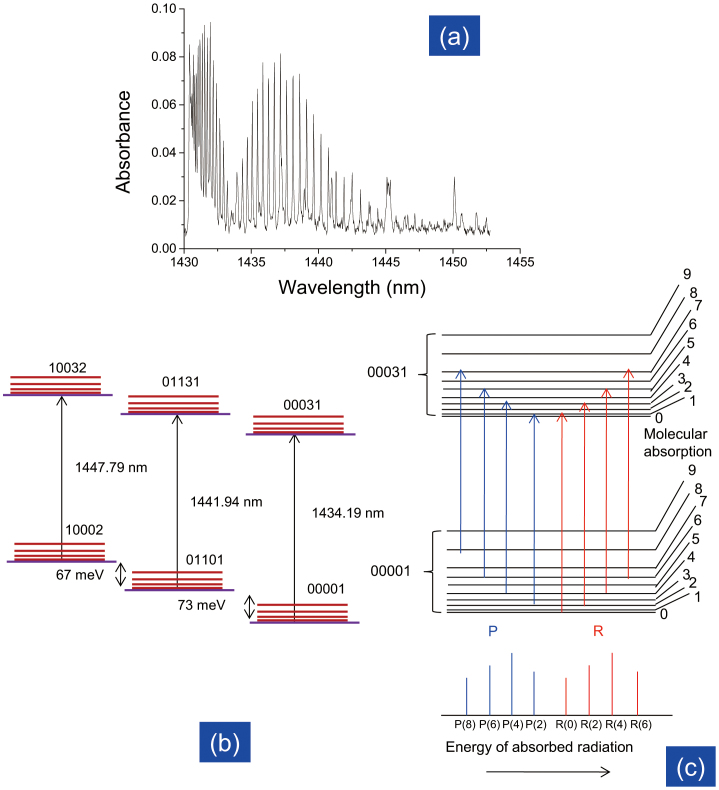
Spectral characteristics of CO_2_. (a) CO_2_ absorption spectrum at 1430–1452 nm. (b) Energy band diagram of the three main transition bands in the wavelength region of 1430–1452 nm: asymmetric vibrational transition 00001 → 00031 and the accompanying hot bands 01101 → 01131 and 10002 → 10032. (c) Schematic diagram of the vibration-rotation spectra of 00001 → 00031 transition band, with the P branch (vibrational transition accompanied by a decrease in the rotational quantum number −1) and R branch (vibrational transition accompanied by an increase in the rotational quantum number +1).

The absorption spectrum of CO_2_ was measured using infrared spectrometer and a halogen lamp as a light source. To determine the CO_2_ concentration, first, the intensity of transmitted light (*I_0_*) was measured without CO_2_ gas in the chamber. Later, CO_2_ was added to the chamber and the intensity of transmitted light was measured (*I*). The total absorption by CO_2_ was calculated using the equation for the absorbance 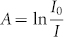. A typical measured spectrum is shown in Fig. 1(a).

The absorption was measured in the wavelength region from 1430 nm to 1452 nm, where the absorption spectrum peaks originate from the asymmetric stretching of CO_2_ molecules. We confirmed that this spectrum is similar to the one recorded in the HITRAN (High-Resolution Transmission Molecular Absorption) 2004 database. The spectrum in this region is dominated by the 3*ν*_3_ overtone 00001 → 00031, the accompanying hot band 01101 → 01131, and another hot band 10002 → 10032^14^. The details of these three bands obtained from calculations are listed briefly in Table 1^15^. The 00001 → 00031 band has a band centre at 1434.19 nm, the accompanying hot band 01101 → 01131 has a band centre at 1441.94 nm, and another hot band 10002 → 10032 has a centre at 1447.79 nm. The line strength of the 01101 → 01131 hot band is about 1.25 × 10^−22^ cm^−1^/molecule-cm^−2^, which is 10 times smaller than that of the main band (1.45 × 10^−21^ cm^−1^/molecule-cm^−2^). The line strength of the 10002 → 10032 hot band is about 2.69 × 10^−24^ cm^−1^/molecule-cm^−2^, which is 1000 times smaller than that of the main band. The corresponding energy band diagram of these three transition bands is illustrated in Fig. 1(b).

According to Table 1, the 00001 → 00031 band ranges in wavelength from 1430.89 nm to 1468.65 nm and consists of numerous spectral lines resulting from the vibrational and rotational motion. The vibration-rotation spectra of CO_2_ for the 00001 → 00031 transition have been studied in detail previously. Table 2 shows the line positions taken from the work of C. Miller^14^,^16^. The lines are mostly divided into two branches, the P branch and R branch. The R branch results from a vibrational transition accompanied by an increase in the rotational quantum number (+1), and the P branch results from a vibrational transition accompanied by a decrease in the rotational quantum number (−1). The concept behind the P branch and R branch is illustrated in Fig. 1 (c). The line positions in the spectrum measured in our experiment are summarized in Table 2, along with the reported values *k*′ values for comparison, and they are found to coincide within an average deviation of 0.06 cm^−1^.

Other than the spectral lines originating from the 00001 → 00031 transition, we also found some weak and irregular lines from the hot band transition, the details of which are summarized in Table 3^17^. We also compare our measured line positions with the values reported for the 01101 → 01131 hot transition^17^. Because the line strength of the other hot transition, 10002 → 10032, was too weak compared to that of the cold band, it was not detectable by our the experimental setup.

### Vibrational energy transfer

**Figure 2:**
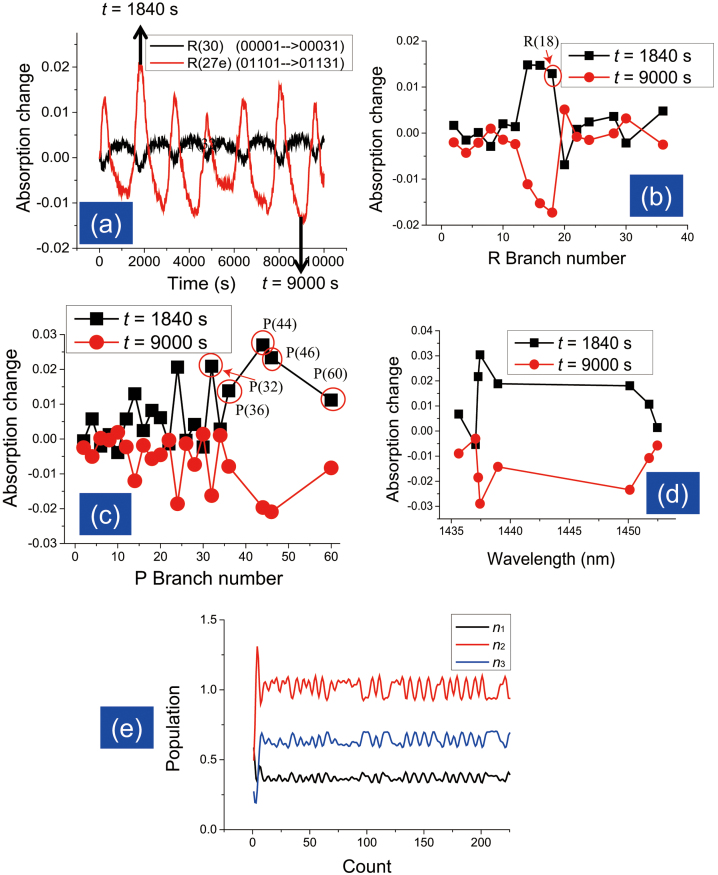
Changes in absorption under UV irradiation. (a) Absorption change at transition band R(30) (cold band 00001 → 00031) and band R(27e) (hot band 01101 → 01131) due to UV irradiation (λ = 213 nm) of CO_2_. The absorption maximum and minimum occurs at *t* = 1840 s and *t* = 9000 s, respectively. (b) Changes in absorption of the R branch for the 00001 → 00031 transition band at *t* = 1840 s and *t* = 9000 sec due to UV irradiation. (c) Changes in absorption of the P branch for the 00001 → 00031 transition band at *t* = 1840 s and *t* = 9000 s due to UV irradiation. (d) Changes in absorption of the hot transition band 01101 → 01131 at *t* = 1840 s and *t* = 9000 s due to UV irradiation. (e) Changes in the populations at the three energy levels *n*_1_, *n*_2_, and *n*_3_ with time, obtained by solving the rate equations.

Next we used a YAG laser of wavelength λ = 213 nm and observed the change in the CO_2_ absorption spectrum at *P* = 40 kPa and *T* = 295 K. All of the absorption peaks mentioned in Tables 2 and 3 were considered. The experimental results indicate that the absorption peaks change periodically with time and have a strong correlation with each other. For example, in Fig. 2(a), we see that the R(30) peak from the 00001 → 00031 transition and the R(27e) peak from the 01101 → 01131 transition oscillate with opposite phases and have a correlation co-efficient of −0.83. Peak R(27e) has its maximum absorption at time *t* = 1840 s and minimum absorption at *t* = 9000 s. We have plotted all the *R* branch and *P* branch peaks of the 00001 → 00031 transition band with respect to the branch number in Figs. 2(b) and 2(c). In addition, the 01101 → 01131 hot band peaks are also plotted in Fig. 2(d). The symmetric nature of the graph suggests that all of the absorption peaks oscillate in the same way, with extreme values at *t* = 1840 s and *t* = 9000 s. This periodic nature of the absorption change indicates that population inversion in the ground states of the transition bands occurs after a certain period of time, which is almost 1000 s in our experimental results.

We consider this change in the spectrum to result from the intramolecular vibrational energy transfer due to collisions between CO_2_ molecules. When sufficient heat is supplied, molecules at lower energy levels can be excited to upper levels, but the oscillating change in the absorption peaks indicates that the energy transfer should be reversible. If the population in the upper energy level becomes sufficiently high, some of the molecules will relax, causing the population to eventually decrease. Furthermore, excitation and relaxation from different energy levels can be considered. Vibrational energy transfer between the ground energy levels of the cold band (00001 → 00031) and the hot band (01101 → 01131) is a well-known phenomenon^18^, equivalent to the 00001 → 01101 bending vibrational transition, which requires an energy of 73 meV (667.4 cm^−1^). Figure 2(d) shows that the hot bands at wavelengths of 1435.63 nm, 1437.28 nm, 1438.95 nm, and 1450.13 nm particularly active, which is evidence of this vibrational energy transfer phenomenon. Moreover, we also observed certain branches of the cold band to be active like the hot bands, such as R(18), P(32), P(36), P(44), P(46), and P(60). This suggests that energy transfer between different P and R branches of the 00001 → 00031 cold band can also be considered.

To analyse the periodic population inversion in the ground states of the transition bands, we also performed rate equation analysis. A simple model with three energy levels is suggested, under the assumption that the excitation and relaxation occur spontaneously. The energy levels are noted as l, 2, and 3 and their corresponding populations are *n*_1_, *n*_2_, and *n*_3_. The rate equations are given below as 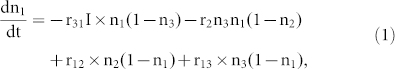

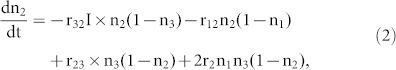

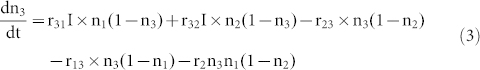
where *r*_ij_ is the transition rate from energy level *j* to energy level *i* and *I* is the light intensity.

To understand the population inversion between the cold band and the hot bands, let us consider energy level *n*_1_ as the ground state 00001, *n*_2_ as the excited state 01101, and *n*_3_ as the other excited state 10002. Vibrational energy transfer between all three states occurs according to the following reactions. 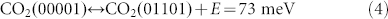

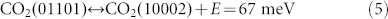

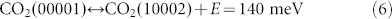

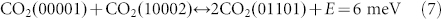


By performing a simulation using the differential rate equations, we solved for the energy level populations *n*_1_, *n*_2_, and *n*_3_. The initial population values were set as *n*_1_ = 1.0, *n*_2_ = 0.0, and *n*_3_ = 0.0, since the molecules exist in the ground state initially. As shown in Fig. 2(e) we find that the populations of all three energy levels oscillate with time, *n*_1_(00001) and *n*_3_ (10002) with the same phase and *n*_2_ (01101) with the opposite phase. The correlation coefficient between *n*_1_(00001) and *n*_2_ (01101) is −0.85, which is almost the same as the experimental correlation coefficient between the absorption changes at R(30) (from 00001) and R(27e) (from 01101), −0.83. Therefore, this three-level system can explain the oscillations observed in absorption peaks under UV irradiation without any nanostructure, supporting the vibrational energy transfer mechanism.

### Phonon-assisted vibrational energy transfer

**Figure 3:**
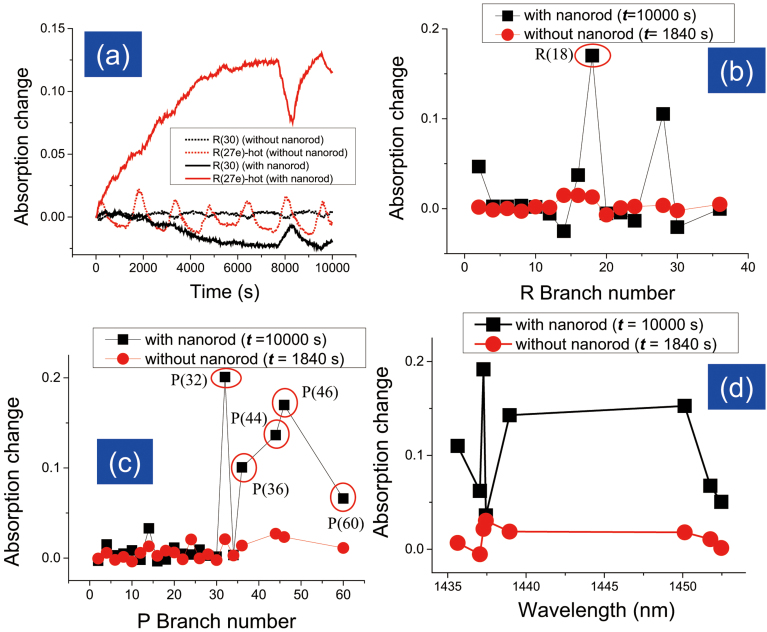
Changes in absorption with and without nanorods. (a) Changes in the absorption at the transition band R(30) (cold band 00001 → 00031) and band R(27e) (hot band 01101 → 01131) due to UV irradiation (λ = 213 nm) of CO_2_. Bold lines were obtained for irradiation of the nanorods, and dotted lines were obtained for irradiation without nanorods. (b) Changes in the absorption of the R branch for the 00001 → 00031 transition band due to UV irradiation, with (at time *t* = 10000 s) and without using nanorods (at time *t* = 1840 s). (c) Changes in the absorption of the P branch for the 00001 → 00031 transition band due to UV irradiation, with (at time t = 10000 sec) and without nanorods (at time *t* = 1840 s). (d) Changes in the absorption of hot transition band 01101 → 01131 due to UV irradiation, with (at time *t* = 10000 s) and without nanorods (at time *t* = 1840 s).

To understand the effect of the DPP, we irradiated ZnO nanorods with λ = 213 nm light and observed the changes in the CO_2_ spectrum. The nanostructures were found to induce significant changes in some absorption peaks. Figure 3(a) shows the absorption changes at R(30) peak from the 00001 → 00031 transition and at the R(27e) peak from the 01101 → 01131 transition, which have a correlation coefficient of −0.97. Instead of the oscillating nature of the absorption change observed under UV irradiation without nanorods, we see the absorption at the R(27e) peak increasing and that at the R(27e) peak decreasing continuously with time, and both saturate after about *t* = 6000 s. The R and P branches of the 00001 → 00031 transition band are plotted as a function the branch number in Figs. 3(b) and 3(c), and the 01101 → 01131 hot band peaks are plotted in Fig. 3(d). The changes in absorption without nanorods at time *t* = 1840 s are also plotted in Figs. 3(b), 3(c), and 3(d) so that the nature of the absorption changes can be compared. As was the case for irradiation without nanorods, the peaks that are particularly active are R(18), P(32), P(36), P(44), P(46), and P(60) from the 00001 → 00031 transition and the hot bands at wavelengths of 1435.63, 1437.28, 1438.95, and 1450.13 nm. For irradiation with nanorods, however, the population increases at these particular bands are significantly higher than those at the other energy bands. While the populations at the different P and R branches were quite uniform during irradiation without nanostructures, irradiation of the nanostructure results in irregular population increases in these particular branches. Because the R(18), P(32), P(36), P(44), P(46), and P(60) branches require high energies to get excite, the excitation of the DPP must be responsible for excitation of these particular branches.

## Discussion

Oscillation of the absorption peaks is observed regardless of the location of the UV irradiation in the chamber. Moreover, the absorption spectrum is measured for all gas molecules inside the chamber (total number of molecules, *N* = 8.4 × 10^21^). Although the collisional energy transfer between two molecules is very quick, with a lifetime on the order of microseconds, it can be said that energy transfer in the whole system of *N* = 8.4 × 10^21^ molecules takes a much longer time, causing the population inversion occurring after almost *t* = 1000 s.

**Figure 4:**
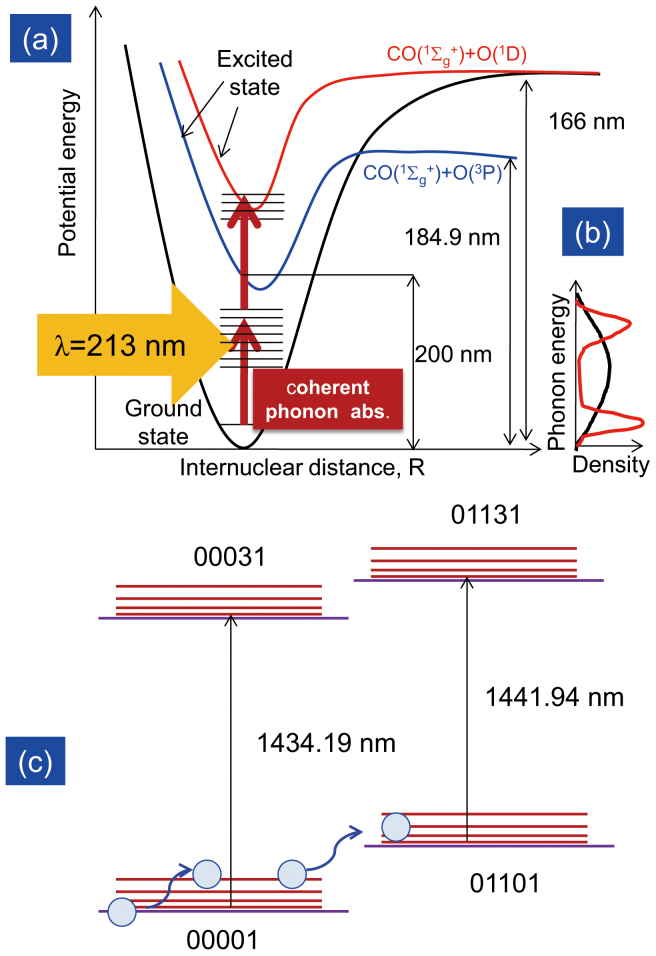
Illustration of phonon-assisted energy up-conversion process. (a) Potential energy diagram for CO_2_ and density of phonon energy states. (b) Density of phonon energy states. Black dashed curve: conventional density of phonon energy states originating from heat absorption. Red solid curve: phonon mode renormalization originating from the coherent phonons absorption. (c) Energy band diagram of the two transition bands. Absorption by CO_2_ molecules of multimode coherent phonons created by DPP excitation leads to a significant irreversible population increase in the high-energy bands, especially hot bands (01101 → 01131) from the asymmetric vibrational transition.

To further support our assumption that intermolecular energy transfer is responsible for the observed absorption changes, we considered other possible mechanisms as well. Because the absorption wavelength of the CO_2_ molecules has been reported to be smaller than 200 nm, it is impossible for the molecules to reach the excited state by absorbing radiation at λ = 213 nm (Fig. 4(a)). Therefore, only vibrational energy transfer at the electronic ground state needs to be considered. Moreover, neither of the product molecules of CO_2_ dissociation reaction, CO and O_2_, would show infrared absorption in the 1430–1452 nm range: CO absorbs at wavelengths less than 1200 nm and at 1570 nm, while O_2_ absorbs at wavelengths less than 1300 nm and greater than 1560 nm. Therefore, molecular dissociation can also be excluded in this system.

Irradiation of nanorods with incident light at a wavelength of λ = 213 nm causes (1) irreversible vibrational energy transfer from the cold bands to the hot bands (Fig. 4(c)) and (2) a significant population increase at specific high-energy transition bands. The DP is known to be able to excite multimode coherent phonons inside the nanostructure, which can be absorbed by molecules around the nanostructure (Fig. 4(a)). The coherent phonon absorption due to the continuous irradiation with UV light causes the rate of excitation of the molecules to the upper energy level to be significantly higher than the relaxation rate. While absorption of phonons from heat energy produces a uniform population distribution as shown in Fig. 2(c), the non-uniform population distribution observed in this experiment as shown in Fig. 3(c) can only be explained by the absorption of coherent phonons. This phonon-assisted energy up-conversion process can be explained as CO_2_ phonon mode renormalization (Fig. 4(b))^19^. The heat absorption results in a broad density distribution of phonon energy states (dashed black curve in Fig. 4(b)), while absorption of multimode coherent phonons by CO_2_ molecules created by DPP excitation leads to a change in the density distribution of phonon energy states to form peaks (red solid curve in Fig. 4(b)). This hypothesis is supported by comparing Figs. 2(c) and 3(c), in which the distributions of the absorption changes are uniform and non-uniform, respectively. In particular, the absorption change at the P(32) peak in Fig. 3(c) was 7 times greater than that in Fig. 2(c), indicating that the phonon energy states were renormalized. This process can also be used to induce absorption and dissociation of CO_2_ with wavelengths longer than the absorption and dissociation wavelengths, which could make it possible to dissociate CO_2_ using the solar spectrum.

In summary, experiments on the effect of the DPP on CO_2_ molecules during ultraviolet irradiation have been conducted. In particular the CO_2_ absorption spectrum was measured in the near-infrared region (wavelength 1430–1452 nm) to observe the changes induced by the DPP. Reversible and periodic vibrational energy transfer was observed during irradiation of CO_2_ with incident light with a wavelength of λ = 213 nm, which is greater than the absorption and dissociation wavelengths λ_abs_ and λ_diss_. Rate equation analysis was also performed to support the proposed mechanism and explain the phenomenon. Irradiation of a nanostructure with an average diameter of 50 nm with the same wavelength λ = 213 nm also causes vibrational energy transfer, but in this case the process was irreversible, causing the population at higher energy levels to increase continuously with time. These different characteristics of vibrational energy transfer can be explained using the concept of the DPP. Excitation of a DP along with multimode coherent phonons allows molecules to reach certain high-energy excited states. Absorption of multimode coherent phonons also has the potential to allow multistep excitation and consequently, absorption and molecular dissociation of CO_2_ with incident light with a wavelength of λ > λ_abs_ and λ > λ_diss_. Therefore, photodissociation of CO_2_ using the solar spectrum can be possible. Since Kiss *et al.*^20^ showed theoretically that ZnO is a good candidate for the synthesis of methanol from CO because of the good hydrogen coverage on oxygen vacancies of ZnO, the use of the hydrogen-covered ZnO nanorods will improve the efficiency of CO generation by photodissociation of CO_2_ and eventually result in production of methanol. In this way artificial photosynthesis could be realized using the solar spectrum.

Such a DPP-assisted photodissociation process can also be enhanced when the structure is fabricated using a DPP-assisted fabrication process^12^. Therefore, further increases in photodissociation of CO_2_ are expected if ZnO nanorods are synthesized using a DPP-assisted process. Recently, Wada *et al.*^21^ directly observed DP generation with coherent phonons in homojunction-structured Si light-emitting diode. In this study, the modes of the coherent phonons were determined using pump-probe spectroscopy. In addition, the side band originating from the coherent phonon excitation was observed in the spectra of a Si LED fabricated using a DP-assisted annealing process^22^. By applying this technique to the ZnO nanorod fabrication process, an optimum morphology can be achieved.

## Methods

### Fabrication of nanostructures

**Figure 5:**
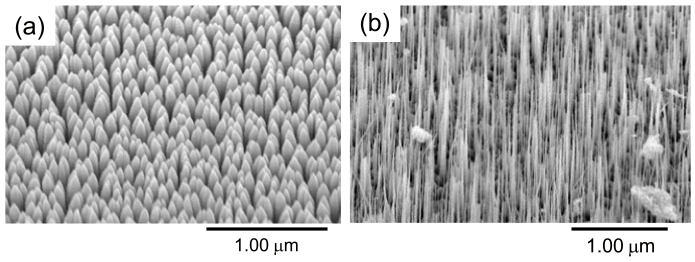
SEM images: (a) ZnO nanorods with average diameters of 50 nm were grown by MOCVD with a growth temperature of 450°C for 30 min. (b) ZnO nanorods with smaller average diameters of 20 nm were grown with a growth temperature of 450°C for 30 min and at 750°C for 10 min.

ZnO nanorods are grown on a sapphire substrate using a catalyst-free metal-organic vapour phase epitaxy (MOVPE) system^23^. Diethylzinc (DEZn) and oxygen are used as the reactants, with argon as the carrier gas. The pressure inside the reactant chamber is maintained at 5 Torr. The substrate temperature is controlled using a thermocouple and a radio-frequency-heated carbon susceptor. The temperature of the system can be adjusted to control the growth rate and structure of the nanorods. We used a two-temperature growth method, with lower-temperature growth of vertically aligned thick ZnO nanorods and subsequent higher-temperature growth of vertically aligned ultrafine ZnO nanorods. In the first step, vertically aligned thick nanorods are grown at 450°C for 30 min. In the second step, ultrafine nanorods are grown at 750°C for 10 min at the tips of the preformed thick nanorods. The average diameters of the nanorods are 20–50 nm. Scanning electron microscopic (SEM) images of the fabricated ZnO nanorods are shown in Fig. 5.

### UV irradiation

**Figure 6:**
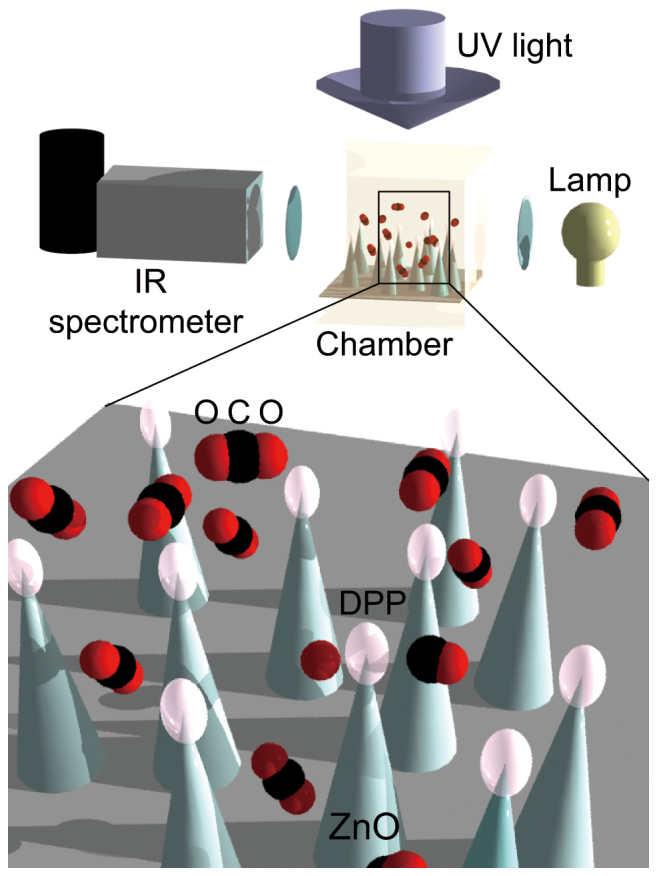
ZnO nanorods were irradiated inside a chamber filled with CO_2_ gas at pressure *P* = 40 kPa, temperature *T* = 295 K, and the infrared absorption spectrum of CO_2_ was measured simultaneously.

We used a 30 cm long glass chamber and filled it with CO_2_ gas at a pressure of 40 kPa, then irradiated the gas with UV light (see Fig. 6). To determine the effect of the phonon-assisted process, we put a substrate with ZnO nanorods inside the chamber filled with CO_2_ and irradiated the nanorods with UV light. The absorption spectrum was measured simultaneously. The spectra were recorded continuously in 1000 shots, where each shot had an exposure time of 10 s.

To interpret these results, let us assume that the intensity of transmitted light at time *t* = 0 is *I*_1_ and that at time *t* is *I*_t_. The concentration of CO_2_ at *t* = 0 is *N* and that at *t* is *N* + Δ*N*, where Δ*N* is the change in concentration over *t* s. According to the Beer-Lambert law, I_1_ = I_0_e^−σLN^ and I_t_ = I_0_e^−σL(N+ΔN)^. From these two equations, we can determine 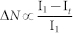.

## Author Contributions

T.Y. planned the project. N.T. performed experiments. T.K. was responsible for providing guidance for the experiments. All authors discussed the results. N.T. and T.Y. wrote the manuscript and edited the final manuscript. All authors reviewed the manuscript.

## Figures and Tables

**Table 1:** Details of the 3ν_3_ overtone vibrational transition bands

Band center (nm)	Vibrational band	Min. position (nm)	Max. position (nm)	Vibrational band strength (cm^−1^/mol. cm^−2^)	Range of rotational lines
1434.19	00001 → 00031	1468.65	1430.89	1.45 × 10^−21^	P(98) → R(98)
1441.93	01101 → 01131	1471.79	1438.56	1.25 × 10^−22^	P(89) → R(89)
1447.78	10002 → 10032	1472.14	1444.30	2.69 × 10^−24^	P(78) → R(78)

**Table 2:** Line positions of the 00001 → 00031 band

Line	λ (nm)	*k* (cm^−1^)	*k*′ (cm^−1^)	Line	λ (nm)	*k* (cm^−1^)	*k*′ (cm^−1^)
R(36)	1430.92	6988.501	6988.501	P(8)	1435.48	6966.301	6965.817
R(30)	1431.08	6987.754	6987.604	P(10)	1435.89	6964.331	6963.943
R(28)	1431.2	6987.129	6987.247	P(12)	1436.29	6962.363	6961.995
R(24)	1431.38	6986.255	6986.084	P(14)	1436.75	6960.148	6959.973
R(22)	1431.56	6985.382	6985.428	P(16)	1437.18	6958.066	6957.878
R(20)	1431.77	6984.386	6984.7	P(18)	1437.66	6955.738	6955.709
R(18)	1431.94	6983.513	6983.896	P(20)	1438.12	6953.532	6953.467
R(14)	1432.2	6982.27	6982.068	P(22)	1438.62	6951.087	6951.151
R(12)	1432.43	6981.154	6981.043	P(24)	1439.13	6948.647	6948.762
R(10)	1432.68	6979.911	6979.943	P(26)	1439.66	6946.089	6946.299
R(8)	1432.96	6978.542	6978.77	P(28)	1440.19	6943.533	6943.763
R(6)	1433.27	6977.057	6977.523	P(30)	1440.79	6940.617	6941.153
R(4)	1433.55	6975.69	6976.202	P(32)	1441.02	6939.524	6938.469
R(2)	1433.7	6974.96	6974.808	P(34)	1441.93	6935.164	6935.713
R(0)	1433.96	6973.71	6973.339	P(36)	1442.43	6932.745	6932.884
P(2)	1434.36	6971.731	6970.998	P(40)	1445.14	6919.745	6920.832
P(4)	1434.72	6970.001	6969.345	P(46)	1445.37	6918.668	6917.636
P(6)	1435.1	6968.151	6967.618	P(60)	1450.13	6893.576	6893.221

λ: measured wavelength. *k*: measured wavenumber. *k*′: wavenumber reported in Refs. 14, 16.

**Table 3:** Line positions of the hot band transition (01101 → 00031)

Line	λ (nm)	*k* (cm^−1^)	*k*′ (cm^−1^)
	1435.63	6965.564	
	1437.06	6958.676	
	1437.28	6957.572	
	1437.44	6956.836	
R(27e)	1438.95	6949.502	6949.582
R(3e)	1441.3	6938.19	6938.076
P(36f)	1450.13	6895.938	6895.417
P(41e)	1451.77	6888.153	6888.163
P(43e)	1452.46	6884.862	6885.09

λ: measured wavelength. *k*: measured wavenumber. *k*′: wavenumber reported in Ref. 17.
